# Correlation of HER2/neu antibody response to clinical response in a Phase II trial of the ae37+gm-csf her2 peptide vaccine

**DOI:** 10.1186/2051-1426-2-S3-P90

**Published:** 2014-11-06

**Authors:** Julia Greene, Erika Schneble, Jonathan Martin, Maddie Flores, Alfred Trappey, John Berry, Timothy Vreeland, Diane Hale, Guy Clifton, Sonia A Perez, Michael Papamichail, George Peoples, Elizabeth Mittendorf, Sathibalan Ponniah

**Affiliations:** 1 San Antonio Military Medical Center, Fort Sam Houston, TX, USA; 2 MD Anderson Cancer Center, Houston, TX, USA; 3 Cancer Immunology Immunotherapy Center, Saint Savas Cancer Hospital, Athens, Greece; 4 Cancer Vaccine Development Program, Uniformed Services University of the Health Sciences, Bethesda, MD, USA; 5 Uniformed Services University of the Health Sciences, Bethesda, MD, USA

## Background

We are conducting a Phase II clinical trial of the HER2 peptide vaccine AE37+GM-CSF for prevention of breast cancer recurrence in disease-free, node-positive or high-risk node-negative patients, who have completed standard of care therapy. AE37, an Ii-Key hybrid of the HER2/*neu *derived peptide AE36 (aa:776-790), is an MHC Class II epitope capable of stimulating CD4^+ ^helper T cells. Here, we examine the relationship between HER2 antibody response (AR) and clinical recurrence (CR).

## Methods

Patients with any level of HER2 expression (IHC1-3+) were randomized to receive six monthly intradermal inoculations of AE37+GM-CSF or GM-CSF alone (controls) during the primary vaccination series (PVS) then four booster vaccinations administered every six months. Prior to vaccination (R0) and after the PVS (R6), serum samples were tested for AR against 178 overlapping 17-mer peptides spanning the entire HER2 protein molecule. Using flow cytometry, the percentage of anti-HER2 specific antibodies was measured and expressed as mean fluorescent intensity (MFI). Sub group analysis was completed on HER2 over-expressers (OE) (IHC3+) versus non over-expressers (nOE) (IHC1-2+) and hormone receptor positive (HR+) (ER or PR+) versus HR- patients. Data are means and compared using a chi-square and students t test as appropriate.

## Results

Of 298 enrolled patients, 153 were vaccinated while 145 were randomized to the control arm. Ten nonrecurrent (NR) and five recurrent (R) vaccinated patients were selected and matched for clinico-pathologic factors. HER2 AR was less in NR versus R at R0 (3659mfi ± 70 vs 4799mfi ± 170, p < 0.01) and R6 (3832mfi ± 82 vs 4832mfi ± 30, p < 0.01). ΔR6 (R6-R0), AR change post-PVS, was increased in NR compared to R patients (174mfi ± 36 vs 33mfi ± 70, p < 0.07). AR was greater in HER2 OE compared to nOE (R0: 4991mfi ± 169 vs 3563mfi ± 70, R6: 4721mfi ± 127 vs 3888mfi ± 84, p < 0.01). nOE exhibited larger ΔR6 HER2 AR (325mfi ± 36 vs -269.96mfi ± 69, p < 0.01). AR was greater in HR+ compared to HR- at R0 (4214mfi ± 91 vs 3336mfi ± 62, p < 0.01) and R6 (4448mfi ± 86 vs 3037mfi ± 54, p < 0.01) with HR+ patients exhibiting larger ΔR6 HER2 AR (233mfi ± 40 vs -299mfi ± 36, p < 0.01).

## Conclusions

In a prospective Phase II trial of a CD4+ -eliciting HER2 peptide vaccine, AE37+GM-CSF, patients who recur have higher levels of pre-existing antibodies and lower levels of antibody induction. This suggests that induction, not amplification, of an immune response may be important. Significantly higher AR change post-vaccination in HER2 nOE and HR+ patients suggests benefit of vaccination in less aggressive disease.
